# Spreading Influenza

**Published:** 1919-06

**Authors:** 


### Spreading Influenza.

Dr. H. E. Bernard, State Food and Drug Commissioner of Indiana, has expressed the belief that certain unclean water used in washing dishes may have been the means of spreading

Page(s) missing

